# Supplementary material for: Subcortical evidence for a contribution of arousal to fMRI studies of brain activity
Source: Nat Commun. 2018 Jan 26;9:395. doi: 10.1038/s41467-017-02815-3 (PMC5786066; doi:10.1038/s41467-017-02815-3)
Supplement: Supplementary file 1 — Supplementary Information [file 41467_2017_2815_MOESM1_ESM.pdf]

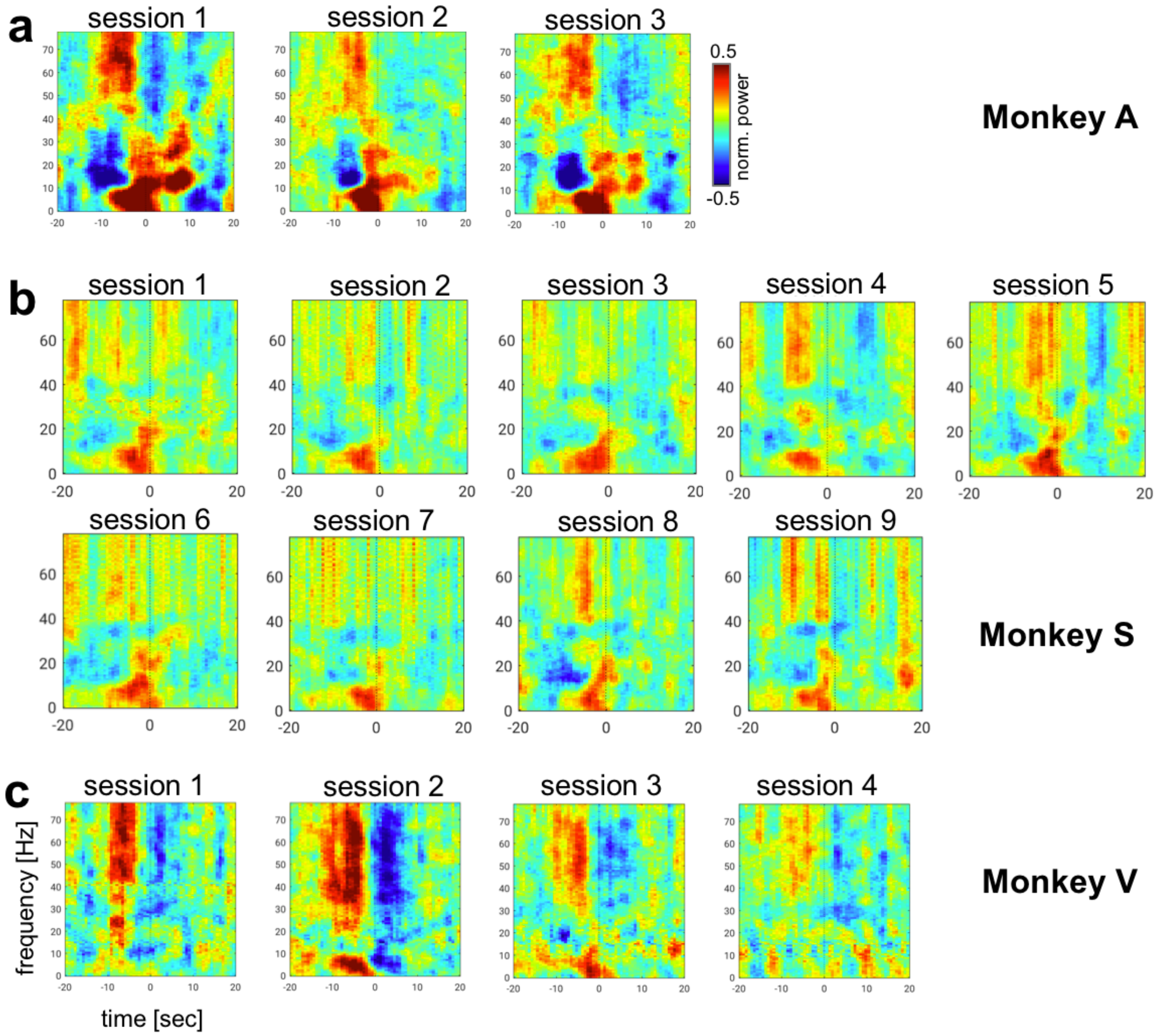


**Supplementary Figure 1**

The fMRI-peak-triggered averaging results in reproducible SST patterns in 3, 9, and 4 sessions acquired on different days from monkey A (a), S (b), and V (c), respectively.

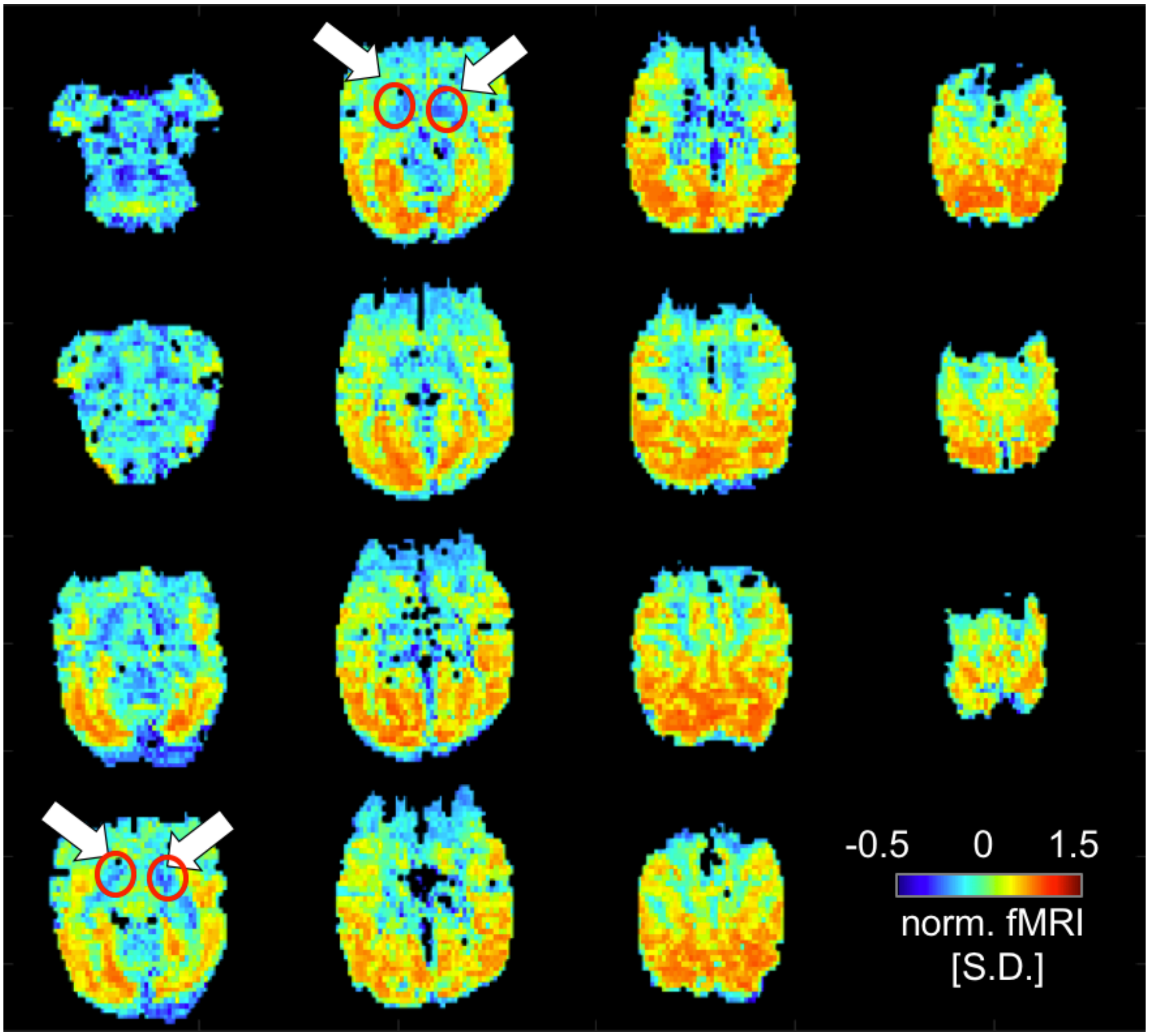


**Supplementary Figure 2**
The global signal co-activation map from a single session of monkey A, which is an average of fMRI volumes at the 30% fraction of time points with the largest global signals. The white arrows and red circles indicate the de-activated regions around the brain areas homologous to the basal forebrain of the human brain.

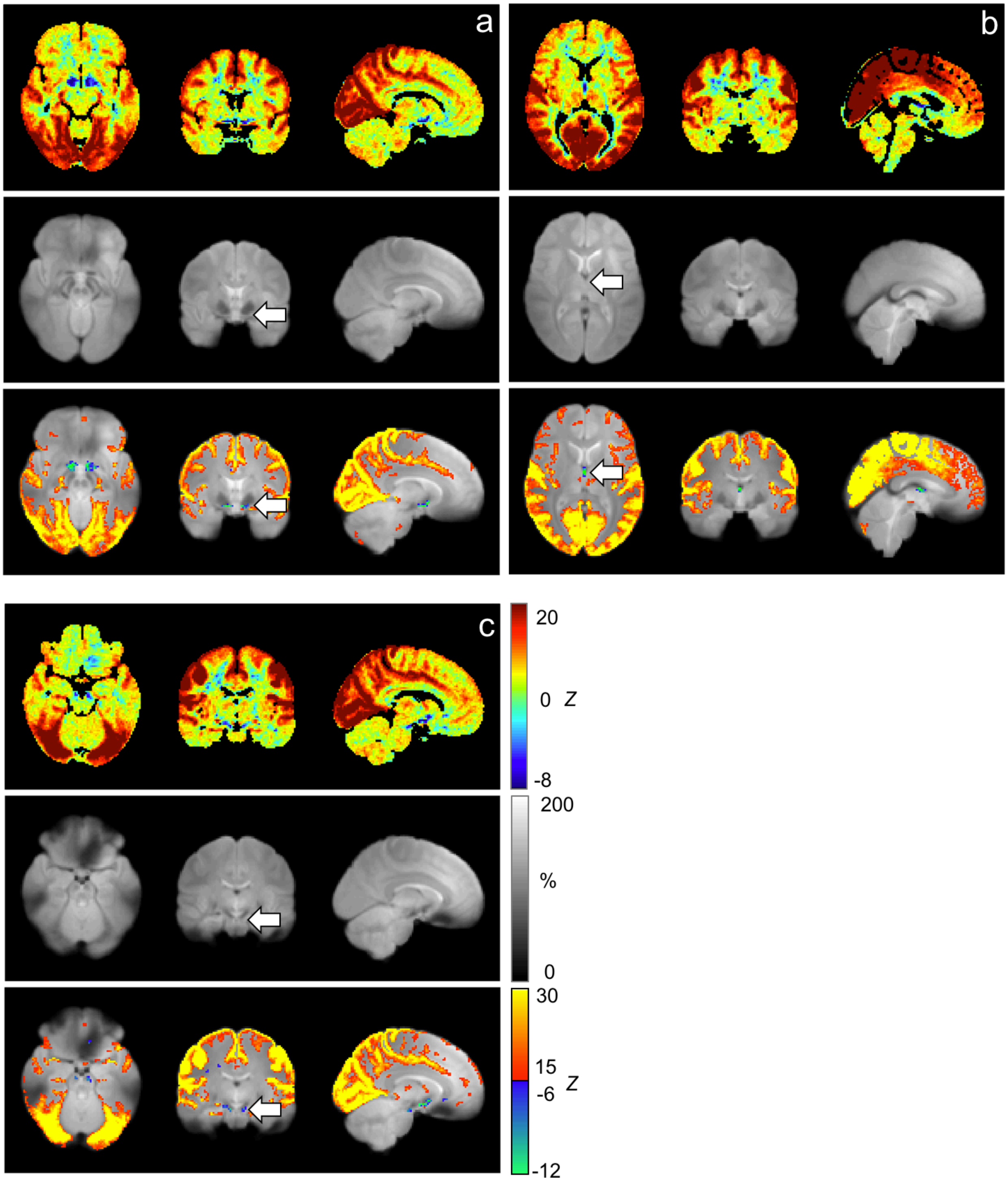


**Supplementary Figure 3**
De-activations in the global co-activation pattern are not an artifact related to magnetic susceptibility related signal loss in corresponding areas. De-activated regions in the global co-activation pattern (top row) are not completely co-localized with brain areas showing relatively weak signal in the average of all fMRI volumes (middle row), and this is even clear with overlaying the former onto the latter (bottom row). There are regions that have lower fMRI

signal but do not show de-activations, e.g., the striatum, as well as de-activated areas showing normal fMRI intensity, e.g., the majority part of the basal forebrain de-activated site. The de-activated voxels in the midbrain structure did show lower mean fMRI signal, but adjacent voxels that also have weak mean fMRI signal show no de-activation at all, especially in a structure right above the de-activated region. The MNI coordinates for slices showing in (a), (b), and (c) are [11, 2, -10], [2, -8, 7], and [-8, -14, -18] respectively. The white arrows in (a), (b), and (c) point to the de-activated regions in the nucleus basalis (NB) of the basal forebrain, the dorsal midline thalamus, and the midbrain structure, respectively. The fMRI intensity map (middle row) is shown as the percentage of the overall mean across the whole brain. It should be noted that the co-activation patterns (top row) and the fMRI intensity map (middle row) focus on very different aspects of the fMRI signals, with the former reflecting relative changes and the latter showing the absolute signal intensity that is typically not analyzed by fMRI studies.
